# Outreach services to improve access to health care in South Africa: lessons from three community health worker programmes

**DOI:** 10.3402/gha.v6i0.19283

**Published:** 2013-01-24

**Authors:** Nonhlanhla Nxumalo, Jane Goudge, Liz Thomas

**Affiliations:** 1 Centre for Health Policy, School of Public Health, Faculty of Health Sciences, University of the Witwatersrand, Johannesburg, South Africa; 2 Medical Research Council - Health Policy Research Group, Pretoria, South Africa

**Keywords:** primary health care, access to care, community health workers, social determinants of health, accountability, South Africa

## Abstract

**Introduction:**

In South Africa, there are renewed efforts to strengthen primary health care and community health worker (CHW) programmes. This article examines three South African CHW programmes, a small local non-governmental organisation (NGO), a local satellite of a national NGO, and a government-initiated service, that provide a range of services from home-based care, childcare, and health promotion to assist clients in overcoming poverty-related barriers to health care.

**Methods:**

The comparative case studies, located in Eastern Cape and Gauteng, were investigated using qualitative methods. Thematic analysis was used to identify factors that constrain and enable outreach services to improve access to care.

**Results:**

The local satellite (of a national NGO), successful in addressing multi-dimensional barriers to care, provided CHWs with continuous training focused on the social determinants of ill-health, regular context-related supervision, and resources such as travel and cell-phone allowances. These workers engaged with, and linked their clients to, agencies in a wide range of sectors. Relationships with participatory structures at community level stimulated coordinated responses from service providers. In contrast, an absence of these elements curtailed the ability of CHWs in the small NGO and government-initiated service to provide effective outreach services or to improve access to care.

**Conclusion:**

Significant investment in resources, training, and support can enable CHWs to address barriers to care by negotiating with poorly functioning government services and community participation structures.

Many low- and middle-income countries face growing health inequities and have made insufficient progress towards the millennium development goals (MDGs). This has been attributed to the poor performance of the health system as well as barriers to care experienced by poor and vulnerable communities. The lack of access to transport, clean water, sanitation, and nutrition limit health improvements. International calls for greater focus on the social determinants of health have highlighted the importance of services capable of responding to the complex intertwined social causes of ill health experienced by marginalised communities (1, 2).

Community health worker (CHW) programmes aim to improve access to care by providing outreach services. Operating at the interface between health systems and communities, CHWs have a crucial role in assisting households to overcome barriers to care. Although there is growing evidence of the effectiveness of CHWs to facilitate improvements in certain health outcomes (3), programmes often fail because of insufficient skills or support (4). Information about successful outreach programmes is needed to guide policy and implementation. This article examines the factors that facilitate success (and failure) of three South African CHW programmes with differing institutional contexts: a small local non-governmental organisation (NGO), a local satellite of a national NGO, and a government-initiated service.

This article provides a brief description of the current challenges faced by the South African government in addressing increasing health inequities as well as current efforts to strengthen primary health care (PHC) through CHW outreach teams. The subsequent sections describe the methods of the study, present and discuss the key findings, and conclude with lessons for CHW programmes.

## Background

South Africa's transition to democracy in 1994 was accompanied by the development of progressive policies in all sectors to address the past structural inequities inherent in the apartheid system and entrench the far-reaching rights in the Constitution (i.e. ‘the progressive realisation of the right to healthcare, housing and education’) (5). Since 1994, South Africa has made considerable investment in PHC through increased infrastructure, rapid expansion of TB, HIV, and maternal-health-related programmatic interventions. This has been coupled with an increase in utilisation of services and the re-emergence of CHWs (6). However, these efforts and investments have not resulted in the expected improvements in the MDGs and other health outcomes due to the complex and growing burden of disease, and the failure to develop and implement an efficient district health system (DHS), responsive to local needs (6). The increasing numbers of service delivery protests by local communities around the country, after 18 years of democracy, demonstrate the frustration of many who have yet to benefit from the provision of basic services.

CHW programmes were initiated in the 1970s by non-governmental organisations (NGOs) in response to the inadequate and intentionally inequitable PHC services under the apartheid government (7–10). Although these programmes have gone through a range of changes, many of the programmes still remain active. In contrast to CHW programmes in countries such as Iran and Brazil, where there are more formalised and structured programmes, those in South Africa remain diverse, and for the most part, fragmented, unstructured, and unregulated (10, 11). These CHW programmes are primarily run through NGO intermediaries. This sector is largely funded by the government. Many of the international and national NGOs and community-based organisations developed in response to HIV/AIDS-focused funding. The NGOs described in this article form part of this diverse sector.

Current health sector reform in South Africa is focused on strengthening the district and sub-district level, including the formalisation and integration of community-based services. In the draft 2011 policy document on strengthening PHC (12), community outreach teams of CHWs led by a nurse will be responsible at the local level for preventative and promotive care, adherence and psychosocial support, with an overall focus on maternal and child health, HIV and TB, and chronic non-communicable disease (6).

## Methods

We used a case study approach to compare the functioning and context of three CHW programmes that provide services in two provinces, namely the Eastern Cape and Gauteng, in South Africa. This allowed us to carry out an in-depth examination of the organisational and contextual factors associated with outreach services.

### Selection of case studies

The database of registered CHW organisations held by the Gauteng Department of Health was examined to identify NGOs in a sub-region of the province that met the selection criteria (currently providing a wide range of services and with more than 15 CHWs). NGOs that met the criteria were interviewed, and final selection was on the basis of willingness to participate in the study. To better understand the reasons for success (and failure), we selected an organisation with a reputation of being well functioning (Eden programme in the Eastern Cape) to compare with the Gauteng programmes. This NGO was chosen as relationships of trust had been built previously with key players.

### Data collection, ethical consideration, and analysis

Key informant interviews (KIIs) with government officials, NGO managers, and key stakeholders collected data on the role of CHWs, the institutional characteristics of NGOs, and management and support mechanisms for CHWs. Daily activities, and experiences of CHWs, including the types of services provided, the strategies employed to negotiate with formal service providers, and the factors that enabled and/or constrained outreach services were recorded in field notes written after participant observations. Three focus group discussions (FGDs) explored CHWs’ perspectives on the management and support they received and their experiences of working with other sectors. A total of 23 interviews and 74 participant observations were conducted with households. Data collection took place during 2010.

Ethical approval was obtained from the Gauteng Department of Health & Social Development and the Committee for Research on Human Subjects at the University of the Witwatersrand. Prior to data collection, informed consent was obtained from all participants. Formal introductory meetings and information sheets were used. All participants were given the opportunity to refuse to be interviewed or observed without prejudice. Names of the programmes and geographical place names described in the article have been replaced with pseudonyms.

Transcripts and field notes were analysed to ascertain the factors that facilitated (un)successful outreach services. Atlas.ti software was used to assist with the identification of *a priori* and emergent themes. The data were compared within, and between, cases, and we returned to the data to confirm emerging themes. Care was taken to identify evidence that diverged from the dominant perspectives. As the Eden programme was considered to be a ‘good example’, efforts were made to ensure that any perspectives critical of the programme were retained within the analysis.

## Results

### Description of the three CHW programmes

#### Case study 1

The Khanya programme was an independent NGO,^1^ initiated by a local community member that relied on funding primarily from the Gauteng Department of Health & Social Development. The organisation aimed to improve general health outcomes, primarily through home-based care (HBC), tracing patients on chronic treatment, and facilitation of support groups. The CHWs were residents of the community they served. CHWs attended the 69-day training workshop provided by the National Department of Health. The curriculum included HBC, TB DOTS, disabilities, child and family health, pregnancy, and preparedness for disease outbreaks (13). Having completed the course, qualified CHWs were paid a monthly stipend. Very limited ongoing CHW training was available, with no opportunity for internal career progression. In addition to the CHWs, the sole staff member was the manager (who initiated the programme) who was responsible for fundraising, operational management, supervision, and mentorship of the CHWs. A government-employed health promoter offered occasional support to the CHWs during their home visits.

#### Case study 2

The Zola programme was established and coordinated by the Gauteng Department of Health & Social Development's HIV/AIDS Directorate and was administered by the local government. It was funded as part of a national government employment generation scheme. The CHWs, recruited from the local community, attended a required 5-day training course on HIV/AIDS, TB and cancer as well as learning about strategies to support the community's access to other services. The CHWs, paid a monthly stipend, conducted door-to-door dissemination of HIV/AIDS-related information, providing advice on how households could access the range of government sectors such as housing, social welfare, water and sanitation. One manager was responsible for supervision and day-to-day running of the programme. Similar to the Khanya case study, the organisation did not offer any internal career progression opportunities for CHWs.

#### Case study 3

The Eden programme, located in the Eastern Cape, was a satellite organisation of a ‘parent’ national child and youth care NGO. Its core objective was to improve child health outcomes in households infected and affected by HIV/AIDS. CHWs, paid a stipend, linked neglected or abused children with health and legal services, and provided day-to-day care for child-headed households. After a community-aligned recruitment process,^2^ the selected CHWs were required to complete 14 training modules, as well as ongoing assessments, over the 2 years. Content of the modules included the basics of child and youth care work, children's rights, behaviour management, and lifespan development. CHWs received extensive supervision and mentorship; mentors focused on their technical skills and well-being, and various managers were responsible for coordinating different aspects of the programme. Internal career progression was encouraged which led to the retention of skilled staff. The case studies are summarised in Table 1.

**Table 1:** Characteristics of the three community health worker programmes

Characteristic	Khanya Programme	Zola Programme	Eden Programme
Location	Periphery of metropolitan area of Johannesburg, Gauteng Province	Periphery of metropolitan area of Johannesburg, Gauteng Province	Rural re-settlement area, Eastern Cape Province
Community served	Predominately migrants from other South African provinces and neighbouring countries	Predominately migrants from other South African provinces and neighbouring countries	Long-standing community; Most households dependent on remittances from household members working elsewhere
Institutional setting	Independent NGO; run by project manager/fund-raiser and project coordinator; under-resourced, with no legitimate office space	Local government-managed programme; under-resourced and using a community house as an office facility; no organisational structure; hands-off management process	Part of a national NGO; the organisational structure has comprehensive managerial activities to support CHW activities at this and other sites. Office based in temporary rooms
Objective of organisation	General health outcomes	HIV/AIDS health outcomes	Child-focused health outcomes
Activities	Home-based care, tracing of defaulters, support group facilitation, identifying other needs – grants, food, birth certificates, identity documents, and water and sanitation	HIV/AIDS-related information dissemination, specific health campaigns; referral to other government services such as Social Development (grants and food parcels), Home Affairs (birth certificates, identity documents)	Address broader issues of children, ranging from health to social problems (including families); referral to other government services; link and accompany them to legal services, social services to access grants and identity documents; social workers for food parcels, grants, foster services, and safe houses of orphans and abused children, provide and supervise daily after school care for children in Safe Park
Funding source	Gauteng DOH	Gauteng DOH (via the Joburg Metro's HIV Directorate)	International funding (PEPFAR)

### The communities served by the three programmes

The Khanya and Zola programmes served communities located in a predominately urban province. The high level of poverty in both communities was exacerbated by unemployment coupled with high levels of chronic and infectious disease. Households often had insufficient food. With poor transport networks, health and social welfare services located at some distance were not easily accessible:

There's only one kombi (bus) that passes through the community; if you miss that kombi, you have to walk to the clinic *…*” (KII –Khanya).

In other examples, lack of food prevented clients with TB from taking their medication and the lack of water for washing deterred clients from attending the clinic. The failure of local government to provide basic services limited the use of the available health care. Nationally provided welfare services, such as social grants and food parcels, contributed to meeting the basic needs of all three communities. However, the clients of CHWs often did not have identity documents and birth certificates required to obtain these social benefits. The struggles of a typical household are described in Box 1.

***Box 1*. A typical household**

During a participant observation, a CHW visited an elderly couple who had been farm employees. Laid off with no formal employment benefits, the couple lived in a makeshift building without water and sanitation. They rely on scraps of food from neighbours, who are equally poor. The elderly woman was too sickly to walk to the nearest clinic. The old man mentioned that at times he walks up to the road to stop a taxi so that it can drive inland to collect the old woman. The CHW inquires if they buy food because there was no sign of food in the house. The elderly man comments that he sometimes does not have the strength to cook because he has to set up a fire outside. (*Field notes: Khanya*).

The community served by the Eden programme was located within one of the poorest provinces in South Africa. The dense rural settlements were established by the apartheid government's separate development policy. Government services are still sparse, inefficient, and inadequately financed. Many of the families, resident in the area for at least two generations, depend on remittances from migrants working in other provinces. As in the Gauteng case studies, poverty is pervasive. Although many of the homes were formal structures (constructed of brick), overcrowding was common: *“*The hygiene in one household where I worked for two years was very bad, because the house was small and there were 14 or 15 people in the house” (FDG – Eden).

### Conceptualising the role of CHWs in each of the programmes

Understanding social determinants as a cause of poor health is key to shaping the role and services of CHWs. The Khanya programme was conceptualised within the health sector and CHW activities were confined to health issues. The Zola programme aimed to provide health information and refer clients to a range of social services. The Eden programme aimed to meet the needs of children affected by the HIV epidemic, which often included responding to broader household needs: “*…* if there is alcohol abuse in the family, it leads to the possibility of being infected with HIV/AIDS so we do early intervention for those families” (FGD – Eden). The Eden programme actively recruited community members who understood the need to respond to the broader range of determinants of poor health. Moreover, the CHWs “received training with other service providers … not only health [police, social development], to help them to address the multifaceted aspects of their work” (KII – Eden).

### Outreach services are resource intensive

The success of the Eden programme was, in part, due to the resources invested in ongoing training, supervision, and mentoring to assist CHWs, particularly in problem-solving skills. The programme provided funds for CHWs to accompany clients to access services, such as health care or to register for a social grant. Mobile phone vouchers enabled the CHWs to keep in contact with supervisors. The clients, often disempowered, required the presence of a CHW at a clinic or government department. If negotiations with government authorities proved to be difficult, the CHWs were able to contact a supervisor by cell phone to intervene.

This support encouraged the CHWs to negotiate with service providers on behalf of their clients:

The parents of a physically abused child were not here in Selby, so we persuaded the doctor to admit the child to hospital for some few days so that we can contact the parents. We could not take the child back to the abusive grandmother. (FDG – Eden)

Investment in training and support for the CHWs increased their own commitment. One CHW described the benefit of a ‘care-for-the-caregiver’ programme:

We spent a week on a retreat away from work, during which we did some grief management work. We are attracted to the work that we do because of our own experiences. The psychologist helped us a lot. We were all together and we were sharing and it opened us up so much. (FGD – Eden)

In contrast, the CHWs in the Khanya & Zola programmes were expected to navigate a complex context with limited support. Both programmes had limited capacity to provide subsequent ongoing training and supervision. CHWs had limited prospects of growing within the organisation or furthering their skills at a tertiary level. The CHWs experienced the same struggles and barriers as the households and were unable to adequately guide or accompany their clients to the referral services:

If there was at least someone who is a bit above us, who has the power or the influence to go to those offices for us, so that we can help people. It would make a difference but we are really standing on our own feet … we are on our own. (FGD – Zola)

### Reporting mechanisms – Monitoring and Evaluation indicators

In the Eden programme, the CHWs reported on the activities and progress of each household on a case-by-case basis. This mechanism ensured that CHWs took a holistic approach to meeting a child's needs. In contrast, the reporting requirements in the Khanya & Zola programmes were limited to numbers of cases and households visited. Given their monthly quotas, the CHWs were not encouraged or able to invest the time necessary to address the multiple needs of their clients.

### Coping with poor cross sectoral coordination

The fragmentation and resultant lack of coordination within and between government departments at all levels was a common and significant constraint to improving access in all three communities. “You can go to National and you see that sectors [housing, water, social development, health] still function as silos, the same for the Province” (KII – Gauteng District Department of Health representative). Most respondents commented on how the lack of coordinated efforts at the higher levels of government made it difficult for CHWs to provide outreach services to communities:

Coordination can only be achieved if the higher levels are coordinated. If those people that design the key performance targets for the specific departments spoke to one another, it would be so much easier to coordinate at the bottom, because the coordination would already have been established and developed. (KII – Regional Department of Human Development representative – Gauteng)

The CHWs in the Gauteng programmes (Khanya & Zola) found it difficult to navigate this fragmentation with the nominal support provided: “Right now we don't know where to send these clients to … we need social worker services” (KII Zola programme). Many of the patients in these two programmes often gave up going to the various government departments. In contrast, the Eden CHWs were able to link clients up with different government departments, despite the limited coordination within and between departments: “The team has formed really good relationships with the police, the school principals, with some of the key people in the hospital and the clinics. These relationships facilitate many referrals” (KII–Eden). It was noted that the programme had limited interaction with the health sector – an exercise which could have enabled the CHWs to respond better to the health needs of their clients rather than solely relying on other health-related NGOs.

### Accountability of service providers and community leaders

The lack of political accountability across all case studies had a detrimental effect on CHWs’ services. Local politicians (called “ward councilors”) are chosen by their political party, rather than the number of votes from local communities, with negative consequences for accountability and development: “the community constantly complains that things don't get done unless you know ‘X’ [person], or you are related to the ward councilor. That is the deep reality which we have in Selby” (KII–Eden).

Despite these limitations, the ward councilor and other stakeholders were found to have played an active role in setting up and supporting the Eden programme:

… we use the ward committee to communicate with the community. For instance when the NGO coordinators wanted to open a Safe Park for children in these locations (townships), they worked hand-in-hand with the ward committees^3^ and with the councilor (FGD–Eden).

This relationship, initiated with the assistance of national office of the NGO, was sustained and the programme continued to benefit from support of the ward councilor and other stakeholders.

In contrast, staff at the Khanya and Zola programmes struggled to involve the ward councilor without success: “Politically, our ward councilor is a (name of political party) councilor, so it's difficult to get him on board. He can't relate to the issues” (KII–Khanya).

Moreover, the CHWs were exposed to the continuous turnover of officials so it was difficult to hold specific individuals, or departments, accountable for poor service delivery. The manager in one of the programmes in Gauteng commented:

I requested [the health promoter] to go to social services to see the person in charge. The person in charge was not known. So we ended up not getting any name and unable to contact anyone. If we struggle in this way, it is even more difficult for clients. (KII–Khanya)

Furthermore, the geographical area served by local government offices varied spatially from one service to another. Due to the lack of alignment of areas of jurisdiction, clients were often told that the office, where they were seeking help, was not responsible for the area that a client resided in. Clients were sent from one area to another, often never obtaining any service at all: “Our referral system here is just not working well …. We don't know where to refer. One minute, you refer patients to the South Clinic [12 km] they say to them: ‘No, you have to go to Hamilton’ [18 kms]” (KII–Khanya).

## Discussion

CHWs in the Eden programme made a difference in the lives of individuals and other family members. The ongoing training equipped them with the skills to respond to children's and their families’ needs as well as to negotiate with service providers. The progress of each client was carefully assessed and regularly monitored. The CHWs in the Gauteng case studies lacked the resources to implement such a model. The manager of the Khanya programme was unable to provide training, mentorship, and support to CHW as well as the overall management. Similarly, in the Zola Programme, although closely associated with local government, training and supervision were neglected.

Internationally, strong evidence shows that well-established supervision and training mechanisms are central to the success of CHWs’ programmes (3, 14, 15). The Brazilian and Iranian successes have been attributed mainly to the quality of the initial and ongoing supervision and training (16, 17). Although the Iranian and Brazilian programmes are both predominantly health focused, they rely on CHWs to provide a wide range of services, including addressing the broad social causes of ill-health (16, 17).

Slow global progress to address the social determinants of health is indicative of the poor governance particularly at a local level (18). In South Africa, the lack of accountability has compromised the envisaged role of ward councilors in addressing the needs of the community. The councilors in Zola and Khanya did little to hold civil servants accountable to provide basic services or to support the programmes in more specific ways. This in turn curtailed the ability of the CHWs to provide effective outreach services. In contrast, Brazil's municipal councils are elected by the community and decision-making processes are shared with non-governmental groups (16).

The success of the Eden programme was due to a range of factors. It received sufficient funding to be able to establish an effective local organisational structure (with mentors, coordinators, and an overall manager). The local office was able to use resources to support the CHW in ways relevant to the local context. The programme managers understood the need to view clients holistically, that the social determinants of ill-health are intertwined, and therefore facilitating access to a social grant (to enable access to transport and food) may be the only way to ensure sustained access to care.

The study highlights the importance of a locally based organisation with capacity and resources to provide an enabling and supportive environment for CHWs. District and sub-district health structures in South Africa struggle to provide adequate facility-based care (19). Under the new policies, without sufficient investment in capacity and sufficient resources to support the outreach teams, the current reforms are unlikely to achieve their objectives. It is also questionable whether the current NGO sector should be seen as an appropriate mechanism to provide support to the outreach teams. South African NGOs in this CHW sector are generally small, with poor management systems. Fragmentation results in the duplication of services which, in some cases, are poorly aligned to national priorities (20). However, the Eden programme provides important relief to a particular community. It is a valuable case study with lessons for both government and NGO-run programmes.

### Limitations of study

The study examined the functioning of the CHW programmes and observed CHW/client interactions. However, users’ perspectives were not ascertained. The use of only one successful case study may have limited our understanding of the factors that facilitated success. The inclusion of additional successful case studies would have provided a more comprehensive understanding.

## Conclusion

To facilitate access to care, and reduce the poverty-related barriers to care, the role of CHW needs to be conceptualised with an understanding of the social determinants of ill-health. The success and sustainability of CHW programmes requires the ongoing commitment of resources, including investment in quality training, supervision, mentoring, and organisational support. In addition, resources are needed to support CHWs to navigate uncoordinated and fragmented government services. Ultimately, strengthening health districts and sub-districts is crucial for effective government-led CHW programmes. The national programme of PHC outreach teams in South Africa is unlikely to achieve its expected outcomes unless there is sufficient capacity to support CHWs to operate effectively at the interface between community and the health system.

## Footnotes

1 Not associated with a larger umbrella organisation.

## Footnotes

2 The CHWs, recruited by community members, were required to have prior experience of relevant community service.

## Footnotes

3 The ‘ward’ is the smallest geographical unit in South Africa; the committee consists of community members and is chaired by the ward councillor.
